# Measurement of IgE antibodies to minor components in eosinophilic esophagitis, peanut allergy, and delayed anaphylaxis to mammalian meat

**DOI:** 10.1186/2045-7022-4-S2-O23

**Published:** 2014-03-17

**Authors:** Anubha Tripathi, Elizabeth A Erwin, Lisa J Workman, Scott P Commins, Thomas AE Platts-Mills

**Affiliations:** 1 Division of Asthma, Allergy, & Immunology; University of Virginia Health System, 409 Lane Road, MR-4 Bldg., Box 801355, Charlottesville, 22908, USA; 2 Nationwide Children's Hospital/ The Ohio State University College of Medicine, Division of Allergy & Immunology, Columbus, USA; 3 University of Virginia Health System, Division of Asthma, Allergy, & Immunology, Charlottesville, USA

## Background

In the investigation of food allergy syndromes, serum assays detecting IgE antibodies (Ab) to food allergens use whole extracts on the solid phase, which contain specific allergen components in varying amounts. The interpretation of results assumes that the causative allergen components are adequately represented in the extract and that the presence and level of specific IgE titer to the whole extract of the relevant food(s) are diagnostic of the syndrome. IgE assays for beef or pork can underestimate the IgE Ab to a minor component of mammalian meat, such as galactose-α-1,3-galactose (α-gal). The inciting food allergens in Eosinophilic Esophagitis (EoE) remain unclear, although IgE Ab to milk, wheat, soy, and peanut are frequently present in low titer.

## Method

IgE Ab to relevant allergens and their components were measured in the sera of adults and children with peanut allergy, delayed anaphylaxis to mammalian meat, or esophageal biopsy-diagnosed EoE. Assay methods included: ImmunoCAP (CAP) using whole and component extracts, CAP assays on serial dilutions (1:2 to 1:8) of sera, and ImmunoCAP ISAC (biochip assay for 112 purified allergens).

## Results

Analysis with ISAC correlated well with results using component-specific CAP assays for both peanut allergens (peanut allergy) and inhalant allergens (EoE). By contrast, no positive results for food allergens were found by ISAC in EoE sera that were positive for milk, wheat, or soy by CAP. Dilution assays showed no change (undiluted value vs. calculated titer) in either peanut allergy sera or EoE sera positive for aeroallergens (dust mite and cat); in contrast, calculated titers up to six times the undiluted value were noted for foods in EoE sera (milk, wheat, and peanut) and in mammalian meat allergy sera (beef and pork). CAP assays for 5 milk components revealed positivity to minor components in 50% of EoE sera. CAP assays for α-gal revealed positivity in 100% of patients presenting with delayed anaphylaxis to mammalian meat.

## Conclusion

Differences in the results of the dilution assays demonstrate that assaying undiluted sera can significantly underestimate IgE Ab levels if the IgE Ab are directed against a quantitatively minor component of the extract on the solid phase. These results strongly suggest that the IgE Ab to milk and other foods in EoE sera are directed against a minor component that has not yet been identified.

